# Dr. Alison's Observations on the Pathology of Scrofulous Diseases, with a View to Their Prevention

**Published:** 1825-01-01

**Authors:** 


*Observations on the Pathology of Scrofulous Diseases, with a View to their Prevention.* By W. P. ALISON, M. D. joint Professor of the Theory of Medicine in the University of Edinburgh.

[Ed. Med. Chir. Transactions, Vol. I.]

THE wide diffusion and untractable nature of scrofulous diseases, together with the discrepancies of opinion respecting their treatment, render any enquiries which promise to elucidate their etiology or pathology, most interesting to the practitioner. Whether the present essay of the learned professor shall "give additional precision and confidence to our notions of the means of preventing them," must be decided by the reader himself, when he has perused the original document or the analysis which we here present him.

As a preliminary step, Dr. Alison notices the erroneous *systematic* doctrine that phthisis pulmonalis is generally confined to the period intervening between 15 and 35. Every practitioner must be in the daily habit of seeing this fatal scourge pervade all ranks and all ages—the *latter* in pretty uniform proportion from youth to senectitude. Dr. Alison, however, thinks that there is a material difference, in general, between the phthisis occurring below 35, and that which occurs in later periods of life—not so much in symptoms as in the intimate pathology of the disease. In the lower orders of society it is found that there is a greater frequency of phthisis beyond the age of 40, than between puberty and the age of 30. In the higher classes, on the other hand, fatal phthisis is rare beyond 40, in comparison with its occurrence between 15 and 30. The reason of this will, he thinks, appear, when we take into consideration the differences observable on dissection, at different ages.

The two great pathological features in the lungs of the phthisical, are hepatization and white tubercles. According to Dr. Abercrombie and others, the latter passes slowly and insidiously into ulceration, without acute symptoms—while the hepatized lung is often the seat of considerable inflammatory action. Thus it happens that, in some cases, the antiphlogistic treatment may be serviceable; while in others, the disorganization advances slowly but steadily, without our having any means of arresting its progress. We agree with our author in believing that hepatization may occur in any habit of body, while the germ of tubercles is probably brought into the world with us.

———— Finisque ab origine pendet!

Our author's observations lead him decidedly to the conclusion that the simple white tubercles, imbedded in the lungs, with little surrounding hardness of the pulmonary structure, occur much more frequently in the young than in the middle-aged or advanced in life, and that a much greater part (often the whole) of the induration in the ulcerated lungs of older people, consists of the hepatized induration or dark-coloured condensation of the proper pulmonary substance. He has seen, as we all have seen, in persons advanced in life, all the usual symptoms of phthisis, as cough, dyspnœa, hectic fever, profuse perspirations, emaciation, repeated hæmoptysis, and a mixture of puriform matter in the expectoration, where it turned out on dissection, that there was no ulceration of the lungs, but much disorganization of the condensed or hepatized kind, with effusions into the bronchia &c.

"If this observation shall be confirmed by others, it may be supposed that the phthisis which is fatal to young persons, is more generally the effect of organic change of structure,—of the deposition of the matter of the white tubercles,—and depends more on circumstances of predisposition; and that that which is so common in older persons of the lower orders, is more to be ascribed to frequent irritation, and to repeated and neglected inflammation, not originally of an unhealthy character." 372.

The means of prevention or of cure, in this last class, must of course be sought in antiphlogistic measures. The phthisis of young persons, on the other hand, our author regards as almost exclusively a scrofulous disease—and our success in its prevention must depend mainly on our knowledge of its causes. This investigation our author divides into two heads—which he thinks it is very important to keep strictly separate from each other. "The first object is to determine *the circum-*

*stances* on which the *scrofulous habit,* or *constitutional predisposition to scrofula* depends, or by which it is increased;—the second, to ascertain *the mode in which scrofulous diseases are excited.*"

1. Our author has nothing new to offer respecting the nature of the external causes of scrofula—but in regard to their *relative importance,* he thinks too much stress has been laid on *hereditary descent,* which is irremediable in all—and on *influence of climate,* which is also irremediable, as affecting the bulk of the community. Too little pains he thinks, has been taken "to determine *the amount* of the agency of *other circumstances,* (well known as predisposing the body to scrofulous disease,) from the cold and wet weather of our winter and spring." It is undoubtedly true, as our author observes, that it is not among those who are *most exposed* to atmospheric influence and vicissitudes, that we see most of the bad effects of the said agents. *They* are fortified, as it were, against such agency, which chiefly exerts its baneful influence on those whose previous condition was such as to favour its operation on the body—particularly those with languid circulation, and feeble constitutions. Thus, old people suffer more from cold weather than the young, although they are certainly much less exposed to it. So with the inhabitants of large towns—they are less exposed to the skies than country people, but suffer far more from such exposures—most probably from their greater debility of constitution.

"Now, what is true of the production of disease in general by exposure to cold, seems to be true of the production of scrofulous diseases in particular: but with these limitations, 1. That scrofulous action appears to be excited almost solely in the earlier periods of life*; 2. That to the production of this kind of diseased action, there appears to be required, besides other conditions, a certain peculiarity of habit, not understood, but manifestly hereditary; and, 3. That the constitutional debility, which disposes to scrofulous disease from cold, appears to be more permanent and habitual than that which disposes to the other diseases resulting from this cause." 381.

It is true that every one who is debilitated, even permanently, does not become scrofulous—nor does every one of strong constitution escape. Still, if it appear that, of a given number, previously weakened by other causes, a much larger proportion becomes affected with some form of scrofula than of an equal

"* This is probably connected with 'the only well established peculiarity in the anatomy of the arterial system in early life;—the much greater absolute size of the capillary branches.'—*Gordon's Anatomy, p.* 61."

number, not so weakened, but otherwise similarly circumstanced, we are entitled to conclude that, *in many cases*, the scrofulous tendency depends in part on, or is much increased by, a state of general debility:—" and it is probably only in so far as it depends on this cause that it is remediable." The facts which seem most decisive as to the connexion of the scrofulous habit with general debilitating causes, are recapitulated by Dr. Alison as follows.

" 1. The differences in the symptoms and progress of inflammation, when scrofulous and when healthy, appear manifestly to indicate in the former case a languid state of the circulation, particularly in the capillary vessels of the diseased part.

" 2. The hereditary disposition to scrofula is chiefly transmitted from parents, and is most observed in children, who show evident marks of constitutional debility in other respects.

" 3. There is no state of the body, as every practitioner knows, in which scrofulous action is so easily excited, as the state of great and often permanent debility, which remains after severe febrile disease, continued fever, small-pox, measles, scarlatina, or which follows the long-continued use of mercury, or accompanies amenorrhœa.

" 4. The season at which scrofulous diseases have been observed to prevail most in this climate, is not that when cold weather has recently set in, and is most productive of disease in general, but the end of winter and the spring; and they are then chiefly observed in those young persons who have manifestly lost strength during the continuance of the cold weather. The gradual diminution of strength in weakly persons during cold weather, is naturally to be expected, (conformably with what we know of the agency of cold on the healthy body, and with other physiological principles,) from the habitual chilling of the surface, and more particularly from the little exercise taken by these persons in such weather. It is commonly stated, likewise, that scrofulous diseases are not so prevalent in the coldest climates as in milder, but moister climates, such as that of Britain, where the temperature, during most of the winter and spring, is from 32° to 45°, and the air usually moist; and it is perhaps to be expected, that such weather will be more weakening and depressing to the constitutions of young persons than colder, but dry and frosty weather; because it will restrain them more from taking exercise, and will allow of less evaporation from the surface during any exercise that is taken. The observations made on this head, however, do not seem to me so decisive as they have been thought; because due allowance has not been made for the circumstance of the more northern countries which have been compared with Britain in this respect, being more thinly peopled, and a much smaller proportion of their population being the inhabitants of large towns.

" 5. The most leading fact in regard to the connection of the scrofulous tendency with debilitating causes, (and one which is of itself sufficient for the most important practical application that can be made of knowledge on this subject,) is the much greater frequency of scrofulous diseases in the inhabitants of great towns, than in the agricultural population of any climate." 383.

Dr. A. quotes numerous writers to prove the infinitely greater mortality in towns than in the country. Thus, in London, one half of the children born attain only two or three years, while in several of the country parishes in Switzerland and Scotland, one half attain the age of 45 years. Mr. Malthus has even quoted authority for the probability of life, in one parish in Switzerland, being as high as 61 years!—These facts sufficiently prove that it is not merely exposure to *cold and wet* (for there is abundance of these in Scotland and Switzerland) which causes scrofula.

It has been remarked by Dr. Davis, (Annals of the Universal Dispensary) that the great cause of death among the children of the lower ranks in the metropolis, is inflammation of the lungs or bronchia, occurring either idiopathically or symptomatically. It is highly probable, as Dr. A. suggests, that the inflammation, in these cases, is rendered intractable and fatal by the weakness of habit which likewise renders them disposed to scrofula. In many of them indeed, the marks of incipient scrofula are found on dissection. Dr. A. was told by one of the physicians of the HOTEL DES ENFANS MALADES at Paris, where upwards of 500 children die annually, and whose bodies are almost uniformly opened, that nearly one half of them presented scrofulous tubercles in some part or other. This is a greater proportion probably than would be found in institutions in this country; but it shews the power of scrofula in great towns, even in the fine climate of France. The deaths under 15 years of age, in the practice of the New Town Dispensary, for two years past, and which Dr. A. thinks may be taken as a fair example of the ordinary causes of mortality among the children of the poor in our great towns, were as follows:—hydrocephalus 40—convulsio 9—febris 3—febris infantum remittens 6—cynanche trachealis 3—pneumonia and bronchitis 18—pertussis 17—variola 32—rubeola 14—phthisis 8—tabes mesenterica 11—sequelæ rubeolæ 13—other minor complaints 25—total 201.

Our author thinks that nearly the whole of the hydrocephalic cases may be regarded as examples of scrofulous inflammation—many of them having shewn other marks of that disease. Several of the cases under the heads of pneumonia, pertussis, &c. are recorded as having shewn marks of scrofula on dissection—in short, 65 out of 201 were decidedly scrofulous. It would probably not be over-rating the scrofulous diathesis then, if we placed one third of the children of the lower orders who die in Edinburgh, as under its influence.

If we suppose two-thirds of the children born in London or Paris, to die under 15, and one-third of these to be scrofulous —then two ninths of those born in the cities die *scrofulous* under 15—" as large a portion as die of *all diseases* under 15 in the parishes of Siwtzerland above-mentioned." 391. Our author quotes numerous medical and statistical reports and returns—satis superque—to prove, what is incontrovertible—the great superiority of the country over towns, in respect to salubrity and longevity. It is not so easy, however, to explain the quomodo, as to prove the quo. Towns and congregated societies produce bad health and short life—but *how* do they do so? And how, in particular, do they generate so much scrofula?

" I am thoroughly convinced," says our author, " from the amount of it that I have seen in families not suffering under any material privations, that it depends much more on want of pure air and exercise than on deficient nourishment. But it is enough for our present purpose to assume, what I presume will not be denied, that it is intimately and probably necessarily connected with the state of debility so conspicuous in children of the lower ranks in large towns, compared with those brought up in the country.

" I apprehend we may go a step farther in pointing out this connection, and assert, that the weakness of system produced, in many cases, by residence in *hot climates*, during childhood and early youth, gives probably a disposition to scrofula, in those who are afterwards exposed to the exciting causes of it during early life. We know, at least, that a great majority of the inhabitants of these climates, both Negroes and Hindoos, are unusually prone to scrofula, when they come to temperate climates, and even suffer from it, in some instances, in their own, where Europeans are nearly free from it." 397.

It is undoubtedly true, that the children of Europeans, born in the East and West Indies, are tender plants, and not a little liable to scrofula when they come to reside in the climate of their forefathers—a fact that tends to corroborate the connexion of general debility with scrofula. Another corroborating fact is seen in the agency of tonics, in the cure or prevention of scrofula. Of these tonics, the most powerful is a residence in the country. " There, as Willan observes, a change of pursuits, a more regular plan of diet and exercise, a more clear and purer atmosphere, the salubrious exhalations from growing vegetables, and the grateful stimulus of their odours, in a short time restore vigour to the body, and firmness and serenity to the mind."

Cold bathing is well known to be a corrector of the scrofulous diathesis, as it fortifies the body against fresh attacks of the disease. The rarity of phthisis among butchers, is another illustration of the efficacy of a tonic regimen in lessening the scrofulous tendency. Medicines also, which improve the state of the digestive organs, and thereby allow more nutriment to be thrown into the system, come really under the head of tonics, though not so classed in systems of materia medica.

"Now, having stated the different facts which prove the intimate connection of the scrofulous diathesis with constitutional debility, we have in fact stated all that has been ascertained in regard to it; beyond the general fact of its being, for reasons quite unknown, much more easily developed, even when all other circumstances are alike, in some persons than in others, and particularly in those whose progenitors have suffered from scrofulous diseases. It is possible, no doubt, that it may not be simply by weakening the body that all the causes I have mentioned dispose it to scrofula; but nevertheless, if we can ascertain, at present, *nothing else that is common to all these causes* but this, that they do weaken the body, we are fully justified in ranking them together, in the mean time, under this head; and in fact I believe we may comprehend all that has been ascertained of the causes by which the disposition to scrofulous disease is produced or increased, lessened or destroyed, under the general proposition, that whatever tends permanently to weaken the body promotes it, and whatever tends really and permanently to strengthen the body counteracts it. By far the most powerful of these last are, habitual exercise, pure air, the application of cold in various forms and with certain precautions, and mental excitation; and the evidence that has been stated on a large scale, seems to me sufficient to entitle us to add, that the action of causes of the latter kind must be both certain and powerful on the whole, although many circumstances may occur to embarrass the practical application of them in individual cases." 403.

2. What has been said, applies only to the formation of the scrofulous habit—"not to the excitation of scrofulous disease."

"In some instances, scrofulous disease shews itself in well-marked inflammation, although of a peculiar character, proceeding from its usual causes, particularly from the long-continued application of cold. But in most cases, in which scrofulous diseases are fatal, the diseased action is in internal parts, and the first symptoms are obscure and equivocal. The chief, and certainly the most characteristic appearances on dissection are tubercles, in different stages of progress; and the question as to the mode of excitation of scrofulous diseases, resolves itself into the question,—How these tubercles are formed, and how the changes in them, which become dangerous to life, are produced?" 403.

It is in this point of view that the question respecting the origin of tubercles is chiefly important. Some pathologists place them to the account of inflammation, while others, as Laennec, Baron, &c. consider their formation as quite independent of that process. The expressions of Dr. Baron are, that tubercles, and the disorganizations to which they lead, "are not the product of any species of inflammation; and that although inflammation may attend their growth, and modify *the symptoms* which they occasion, yet it is very different, both in its origin and consequences, from that which attacks a part unaltered by previous disease, being, *in the former case, the consequence*, and in the latter, the cause of the altered texture." Laennec expresses his opinion more guardedly. He says, that inflammation of the lungs occasionally co-exists with tubercles there, and may probably sometimes become the occasion of their development, in subjects predisposed to their formation; while in other cases, the irritation occasioned by tubercles already existing, may induce inflammation. With these admissions, he still thinks that tubercles most frequently form without previous inflammation—and that when inflammation does co-exist with any tubercular affection, it is most commonly *posterior to it in date.*

"On the other hand," says Dr. Alison, "the opinion which I believe has been on the whole more general, at least in this country, that tubercles are a product of a peculiar kind of inflammation, has been lately maintained with much ability, and perhaps in too unqualified terms, by Broussais. Several of the cases related by him*,—in which young men, previously fit for their duties as soldiers, have been seized with catarrhal or pneumonic symptoms, from a well marked exposure to cold, or an injury on the breast; have passed gradually into the state of phthisis, and died in a few months; and have exhibited, on dissection, the lungs completely studded with tubercles of various sizes, and in various stages,—appear to me to furnish very strong evidence; particularly, as the fact of their having been capable of military duty during the fatiguing campaigns of the French army, up to a given time before their death, furnishes a sort of standard of the state of the vital organs up to that time; but as, in all these cases, there appear to have been considerable marks of inflammation (adhesion and hepatization) as well as the tubercles, found on dissection, the evidence deduced from them can hardly be said to be decisive. It may still be said, that the tubercles had all existed prior to the inflammation from the known external causes; and that the symptoms, during the last few months of the patients' lives, had been the consequence of that inflammation supervening on the tubercles, and perhaps quickening their growth, but not producing them. It must be allowed, that there are few cases, in which tubercles are found on dissection, where this ambiguity does not exist." 407.

* "Hist. des Phlegmasies Chroniques, vol. ii."

Dr. Alison seems to steer a middle course between the extremes of Baron and Broussais. His observations lead him to think that, where there is the constitutional tendency to scrofula, tubercles may form in very different circumstances, and with different degrees of rapidity. He has little doubt "that they often do form without inflammation (of such a character as to be detected by symptoms during life) preceding them;" —and that, in the lungs at least, the inflammation, of which the undeniable marks are so often found along with them after death, has really often been posterior to them in date. But he has also been led to believe that it is not merely, as Laennec states, a possibility, but a real and frequent occurrence, that inflammation, acute or chronic (to which he would add, *febrile action)* however produced, becomes, in certain constitutions, the occasion of the development of tubercles. The cases which have occurred to our author, and in which it has appeared to him nearly certain that tubercles had formed in consequence of inflammation, he arranges under two heads:—The *first* consists of cases in which they did not cause death, and were found, on dissection, in an incipient state, but so immediately succeeding to the symptoms, and so closely connected with, or even passing by insensible degrees into, the undeniable effects of inflammation, that it has appeared to him impossible to suppose that their formation had been independent of it. The first case, which is short and interesting, we shall give without abridgement.

"J. P., æt. 4, of a weak habit, but healthy, was seized, in the beginning of November 1822, with febrile symptoms, headache, and pain of abdomen, attended with tenderness on pressure; which last symptom, however, was not urgent. The febrile symptoms remitted repeatedly, but she continued subject to much pain of abdomen, generally with a loose state of the bowels, till the beginning of December, at which time I first saw her. The symptoms had just then undergone a remarkable change,—the pain of the abdomen was almost gone, but she had a great deal of vomiting, excited by almost every thing she swallowed, cold extremities, small, often irregular pulse, and drowsiness approaching to coma, though without headache or delirium. These symptoms lasted with little change, except progressive emaciation, for ten days; the vomiting then ceased, and she was seized with convulsions, followed by coma and dilated pupils; in which state she lay four days, with occasional returns of convulsion, and died on the 19th December.

"On dissection, the ventricles of the brain were found distended with serum.

"Almost the whole peritoneum, except the covering of the stomach, was covered with an exudation, resembling the coagulable lymph so often thrown out on it by inflammation; except that it was generally of a somewhat yellower colour, and was disposed chiefly, though not entirely, in the form of round tubercular eminences, of various sizes, none so large as a split pea. By the intervention of this matter, numerous adhesions of the liver to the parietes of the abdomen, and bowels to each other, had taken place. In various places, where the exudation was thickest, the peritoneum was thickened, and very vascular. In the pelvis there was an irregular mass, of the same appearance, which hardly adhered to the peritoneum. The ligamenta lata of the uterus were much thickened, and of a deep red colour, and on cutting into them, much thick pus was found between their layers.

"This was manifestly a case of conversion of abdominal disease into hydrocephalus. Had it not been for that conversion, the inflammatory appearances in the abdomen might probably have subsided, the pain of abdomen having almost ceased long before death. The disease would then, I have no doubt, from what I have seen in other cases, in which there were depositions on the peritoneum in precisely similar forms, but of larger size and somewhat firmer consistence, have passed into the form of the tuberculated accretions on the peritoneum described by Dr. Baron; and if this had been fatal at a subsequent period, it would have been impossible to judge whether inflammation had produced the tubercular masses that were found or not;—a point, on which the appearances observed in this early stage of the disease in the present case, left, I believe, no doubt whatever in the minds of the gentlemen who witnessed this dissection." 413.

2. The second case was that of a healthy child, two years of age, uncommonly fat and plethoric, who was seized with measles preceded by a convulsion fit, in the end of May, 1823. Some fever and shortness of breath, with trifling cough continued after the eruption had faded. Our author saw her on the 18th June, when she had quick pulse and warm skin, evening exacerbations, tendency to coma, pupils somewhat dilated, vision imperfect, rolling of the head and drawing up of the legs, grinding of the teeth, tension and tenderness of the abdomen, irregular bowels, dark green fæces. "She had a papular, or rather a tubercular eruption on different parts of the skin, that came out a few days previously." These symptoms continued with little change, and she died suddenly, after a slight convulsion, on the 26th June. On dissection, the internal coat of the stomach was manifestly diseased, very red at some points, at others of a brownish colour and softened, with some abrasions towards the pylorus. The liver was firmer and paler than usual. There were a great number of tubercles, easily rubbed off, on various parts of the peritoneum, all quite solid, though many of them exceedingly minute. There were a few in the substance of the liver—many in the spleen, which was enlarged and hard—several beneath the peritoneal coat of the intestines. A portion of the mesenteric glands was tuberculated—many small tubercles on the surface and in the substance of the lungs—the lower part of the left lung was very vascular and hepatized, and in the middle of the hepatized portion, there was a large irregular tubercle of firmer consistence than the others. The head, strange to say, where the immediate cause of death most probably existed, was not examined.

The following are Dr. Alison's deductions from this case.

"1st. That the state of the child's health, before the attack of measles, seems nearly to preclude the possibility of their having existed then.

"2d. That the child never recovered from the febrile and inflammatory symptoms produced by the measles, and died evidently of their sequelæ, about a month after their appearance.

"3d. That the symptoms before death were inflammatory, and the tubercles found after death, in various places, but particularly in the spleen, the mesenteric glands, and the lungs, were each surrounded with unusual vascularity,—and the large tubercle in the left lung with a considerable extent of hepatization." 415.

We can hardly agree with Dr. Alison in thinking that such an extensive tubercular condition, where some of the tubercles too, were large and consistent, was the work of rather less than a month. That their growth might have been greatly accelerated during the measles and the febrile state that succeeded, we have no doubt, but that they were actually formed, *de novo*, within that period, exceeds our belief. Neither do we think that the health and embonpoint of the child, previously to the measles, afford unequivocal proof of the absence of tubercles in the internal organs. We have opened many bodies, where the cause of death was sudden, and where perfect health preceded the fatal event, and yet where numerous tubercles were found in the lungs. We consider the case in question, therefore, as equivocal, and of little avail in the support of Dr. Alison's doctrine. It is nevertheless interesting, on many other accounts. We shall pass over the third case, as not being very satisfactory, in order to bestow more space on the fourth, which is one of considerable importance.

*Case* 4. D. M., aged 18 months, subject for six months to a discharge of matter from the left ear, and more recently to irregular febrile attacks and diarrhœa, but unaccompanied with any pectoral affection, was admitted to the Public Dispensary on the 11th July, 1823, with measles, the symptoms having commenced a few days before. After the decline of the eruption he continued subject to cough, and did not regain appetite or strength. About the 24th July, he became feverish, and the head appeared affected. He had occasional screaming and vomiting, the cough persisting. On the 1st August, he was comatose—pulse 100, sharp and irregular—pupils dilated—countenance pale—skin cool—body much emaciated—breathing rather short—rattling in the throat. He died on the morning of the 3d.

*Dissection.* There was some effusion of coagulable lymph on the surface of some parts of the pleura pulmonalis. The cellular substance beneath the pleura was in some places emphysematous—and in many places there were small elevations of the pleura, covering little cavities, a part of which only were filled with tubercular matter, in a soft gelatinous state, with a little air. There were a great many tubercles, in the same stage of progress, dispersed through the substance of the lungs.

"Immediately adjoining the tubercles, both on the surface and in the substance of the lungs, there was, very generally, increased vascularity, and a degree of condensation of the pulmonary substance, but this was of so small an extent, that every part of the lungs crepitated in the usual manner, when cut across. The bronchiæ, as usual after the measles, were very vascular, and contained much muco-purulent matter.

"The bronchial glands were enlarged, and sections of them shewed increased vascularity, and points of tubercular matter; and it was quite obvious, on minute examination with the microscope, that from the points where the tubercular matter was distinctly formed, there was a very gradual transition into the portions of the glands that retained the healthy structure, so that it was impossible to say where the healthy, but unusually vascular texture ended, and the tubercular substance began.

"On the peritoneal surface of the diaphragm there was a spot of inflammation, with a deposition of matter exactly similar to that observed in Case 1, disposed in the form of irregular tubercles, beneath the serous membrane.

"There was a considerable effusion of serum under the arachnoid membrane; the brain was very soft; and the ventricles distended with serum.

"In the lower part of the left lateral lobe, the substance of the brain was of a red colour, and quite broken down; and a portion of the pia mater, immediately beneath this, was destroyed by ulceration." 419.

The pia mater, immediately around this spot, presented a curious appearance. It was very vascular, and there was much deposition, in the most vascular portions, of a whitish matter, which appeared on examination to be between the pia mater and arachnoid coat, and to cause an intimate adhesion of them.

"In some places, where the vascularity was most intense, this pia mater had the usual irregular form, and just the usual appearance of an inflammatory exudation; but in many others, it was disposed in little roundish masses, having exactly the same appearance as the tubercles of the lungs, and of the bronchial glands, in the same subject. These smaller deposits were of very various density; some appearing, on examination with the microscope, merely as ill defined nebulæ, dimming the transparency of the membrane; others as well defined, opaque circular prominences; but none of them, either here, or in any other texture except the lungs, had the least appearance of being inclosed in vesicles, or cysts. They resembled, very nearly, the pustules so often seen on the tunica conjunctiva of the eye, particularly in scrofulous persons, in their first stage." 420.

Here they had an opportunity of seeing tubercles in their incipient stage—the child having died in consequence of the disease of the brain and effusion into the ventricles. The particulars in this case which appear to our author to be the most decisive are—

"1. The certainty derived from the information, both of the father and mother, who were questioned separately, and were both very intelligent and attentive, that the child had no pectoral symptoms, previously to the attack of measles, with the usual pneumonic symptoms, four weeks before death, but had suffered ever since then from cough, with some shortness of breathing.

"2. The lungs being found, on dissection, studded with tubercles in their soft incipient state, chiefly contained in small cavities, which they did not completely fill; almost all of them surrounded with unusual vascularity, and slight condensation of the pulmonary substance.

"3. The occurrence of tubercles, in the same state of forwardness, but not contained in vesicles, on a portion of the peritoneum, the bronchial glands, and on the pia mater; and their being equally, in all these places, surrounded with increased vascularity.

"4thly, and chiefly, The incipient tubercles on the pia mater being closely contiguous to, of the same appearance as, and evidently connected with, a larger and more irregular deposition of matter, which had the usual characters of inflammatory exudation on the pia mater—which was connected with manifest effects of inflammation in the adjacent substance of the brain,—and which, if it had occurred alone, I think no one could have hesitated to call a product of inflammation.*" 421.

We cannot spare space for the fifth case. The conclusions to which all the observations under this head seem to Dr. Alison distinctly to point, is, "that in certain constitutions,

"* The origin of the tubercles, in these cases, appears to have been very similar to what has been observed by Magendie, in his minute examination of phthisical lungs. At least, his observations tend to confirm the fact of tubercles, in this texture, frequently appearing, at first, in air-cells which they do not completely fill, and being, in the first instance, surrounded with vascularity.—*Journal de Physiologie*, t. i. p. 82."

flammation of various textures tends to effusion of a whitish or yellowish matter, not in any considerable deposits, but chiefly in small circumscribed masses, more or less completely separated from each other, and that these are a common origin of scrofulous tubercles." It is obvious that Dr. Alison's theory is at variance with the hydatid doctrine of tubercles, maintained by Dr. Baron. We cannot presume to reconcile these contending parties. Sunt arcades ambo.

"II. The second set of cases which I have to adduce on this subject, consists of examples, in which children, previously in good health, or at least unaffected with any pulmonary complaint, have been seized with well-marked inflammatory symptoms, generally from a known cause, certainly adequate to that effect; these symptoms have lasted some time, and been manifestly dangerous to life,—have subsided very imperfectly, —the children have passed into the state of phthisis, and died within a few months; and on dissection, tubercles have been found, in various stages of progress, but with little or no other appearance which could be considered either as the effect of the inflammation, *known to have existed*, or as the cause of death." 425.

*Case* 6. A boy, five years of age, pretty stout, but somewhat rickety, and with a small scrofulous sore on his leg, was attacked in the end of November 1815, with well-marked pneumonic symptoms. While these were recent he was seen by different medical men, was twice bled, and had the usual remedies for such complaint. The febrile symptoms abated, but his breathing continued short, his cough troublesome, and he passed gradually into a state of complete hectic, dying considerably emaciated in the end of January, 1816. On dissection, the lungs were found of their natural spongy texture throughout, and the disease appeared to be confined to the bronchial glands, which were enormously enlarged, and all converted into the usual cheesy or tubercular matter. There was no other disease in the thorax or abdomen. For cases 7, 8, and 9, we must refer to the original, as our limits are drawing to a close. Our author has selected the foregoing instances out of many which have occurred to him, and thinks he may with confidence assert that such cases are by no means rare among the children of the lower orders of society in large towns.

"A healthy child," he observes, "shall be seized suddenly with well-marked pneumonic symptoms, either from cold, or from the contagion of hooping-cough or measles. The symptoms shall be such, as to justify a confident expectation (founded on dissections of children dying in the height of such affections, of which I could produce many examples) that if speedily fatal, they will leave behind them the indications of active inflammation. The urgent danger shall be got over, but the cough and shortness of breath shall continue with progressive emaciation and debility, till death takes place, at the end of some weeks or a few months; and on dissection, there shall appear tubercles in great numbers, and sometimes of a pretty large size, with no condensation of the pulmonary substance, or only a slight condensation of it, just about the tubercles." 431.

In drawing conclusions from such cases, our author does not mean to lay much stress on the circumstance of the patient being previously in health. What he chiefly relies on is, *the certainty* of inflammation, *from a known external cause*, having existed, some weeks or a few months *only* before death, and the patient having been in great danger from it, *and never having recovered* from the symptoms of it;—and the numerous and advanced tubercles being the chief or *only appearance* found on dissection.

"The question is, Whether we are to believe that the inflammation caused the tubercles; or that the inflammation, which certainly existed so recently, and the chief symptoms of which never abated, but went on uniformly to the end, did not cause death, nor leave any consequences perceptible on dissection, but merely co-existed by chance with another affection previously existing, and which ran an independent course?" 433.

We have now given a very full analysis of Dr. Alison's paper, and have enabled our readers to judge for themselves respecting the value of the professor's observations. We will not biass their judgment by any commentary of our own. We may just be permitted, however, to say, that the language is by no means so clear and well constructed as we might naturally expect from a professorial chair. There is room for improvement in this respect, and we have no doubt that Dr. Alison will improve accordingly.

